# Remarkable Improvement in Kidney Function in a 93-Year-Old ESKD Patient Under Home Hospice Care

**DOI:** 10.1155/crin/7796641

**Published:** 2025-04-18

**Authors:** Alster Talia, Beizer Reshit, Azulay Daniel

**Affiliations:** Hadassah University Medical Center, Jerusalem, Israel

## Abstract

This report details the remarkable improvement in kidney function and quality of life in an ESKD 93-year-old male patient under conservative management in Israel. The patient, with previous postrenal obstruction due to prostate enlargement and recurring infections, exhibited significant kidney deterioration, reaching a creatinine level of 7.6 mg/dL and a GFR of 6.7 mL/min/1.73 m^2^ in December 2023. Following numerous hospitalizations and ER visits over the previous year, the patient opted for palliative care, prioritizing life quality over life-extending interventions. Over a 6 month period (December 2023–May 2024), the patient received comprehensive palliative care: nurse visits, bimonthly doctor consultations, and medication adjustments based on biochemical home blood tests. Despite initial severe kidney impairment, the patient's creatinine levels improved to 3.2 mg/dL and GFR increased to 18.2 mL/min/1.73 m^2^. Potassium and phosphorus stabilized, and no further ER visits were recorded. Clinically, the patient showed enhanced cognition, memory, and communication and managed peripheral edema effectively. This case underscores the potential benefits of conservative palliative management in an Israeli elderly patient, a phenomenon that is recognized in the US and Canada more often, but not highlighted in the Middle Eastern elderly population. Coordinated efforts of a skilled palliative care team and supportive home hospice care contributed to the patient's clinical improvement. These findings challenge the notion that is often communicated to Israeli ESKD patients, Hatoum and Sperling, that palliative treatment leads to further kidney function deterioration, suggesting that, in some cases, it can result in improved outcomes in an underreported population.

## 1. Introduction

Data concerning the outcomes of patients with end-stage kidney disease (ESKD) in renal replacement therapy (RRT) compared to conservative management exists in small numbers, mostly in United States–based studies on the local population. With the increasing prevalence of CKD among the elderly worldwide, with comorbidities and impaired functional status, understanding management decision implications is essential. In addition, identifying populations in other areas of the world that may be candidates for conservative management is important.

Scarce definitive data on whether dialysis offers better life quality and quantity cause variability in management. United States' nephrologists in recent years signaled the need for a stronger system commitment to building care models supporting various treatment decisions, including the decision to forgo dialysis [1]. Nephrology associations worldwide aim to develop frameworks and, potentially, guidelines to assist in clinical practice [2, 3]. These guidelines are not, however, geographically specific and rely on existing data that are most often United States or Canada-based.

A 2011 review of data on palliative decision-making in ESKD among elderly patients noted that while studies suggested dialysis could extend life in some cases, quality of life benefits remain unclear [4]. Symptom burden and life quality in ESKD managed conservatively was comparable to patients on dialysis in another study in patients [5], while a more recent study found no effect of hemodialysis on symptom burden in terminally ill patients [6]. Several studies suggest that over a certain age (65 or 75, depending on the study), survival rates among patients with CKD may be similar in both treatment strategies after correcting for comorbidities [7, 8].

All data on this topic recommend keeping patients informed about therapy options and symptom management strategies, with the aim of promoting patient values and preferences, shared decision-making, and focusing on improved symptom management. This report presents an elderly holocaust survivor opting for conservative therapy for ESKD and the outcomes over 6 months of follow-up and management with a home hospice program.

## 2. Case Presentation

**Figure 1:**
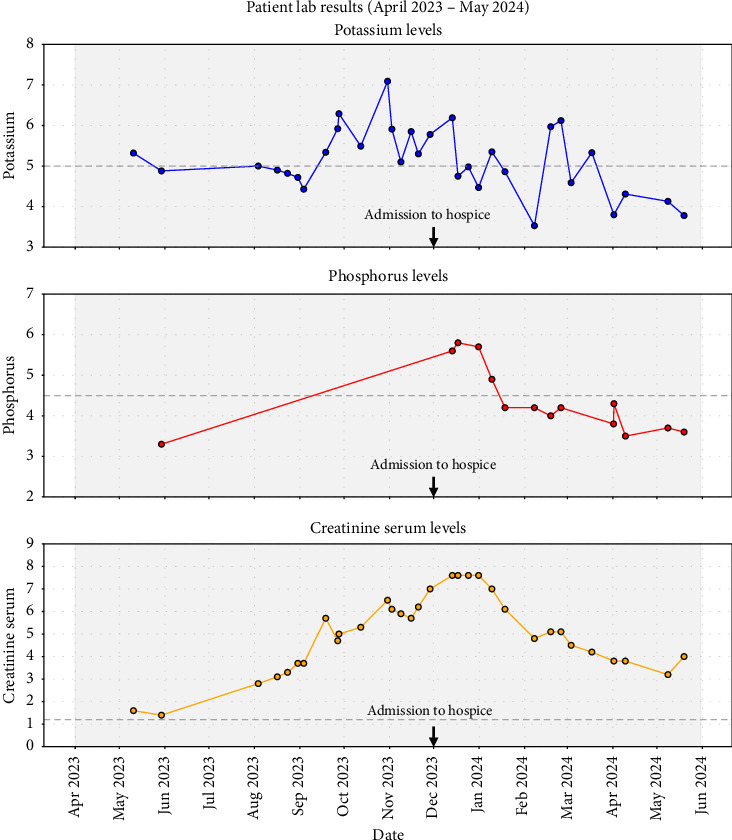
Patient's laboratory results.

We report a 93-year-old male holocaust survivor, living in Israel with ESKD due to postrenal obstruction resulting from prostate enlargement and recurrent catheter-associated urinary infections. Kidney deterioration was first documented in May 2023 with creatinine 1.6 mg/dL (see Figure 1). Despite indwelling urinary catheterization and prostate artery embolization, kidney function gradually declined, reaching 7.6 mg/dL (GFR 6.7 mL/min/1.73 m^2^) in December 2023 when he was admitted to home hospice.

Over 12 months preceding hospice admission (January 2023–December 2023), the patient visited the hospital 12 times in total. Out of these, he was hospitalized five times, either in the internal medicine ward or the urological ward. The number of days in the hospital ranged between 1 day (e.g, during an ER visit that did not result in hospitalization) and 14 days. The causes for hospital admission included acute urinary tract infections (in 7 out of 12 visits), urinary catheter obstruction (in 4 out of 12 visits), and other causes such as acute anemia, deterioration in kidney function, and macrohaematuria (see Table 1). Kidney-related laboratory results deteriorated gradually over these months.

On the last hospitalization (Dec. 2023), GFR reached 6.7 mL/min/1.73 m^2^, potassium peaked at 6.1 mEq/L, and phosphorus was 6.2 mg/dL (see Figure 1). The patient and family were informed about treatment options by nephrologists, and after discussion, the patient opted for conservative management under home hospice. He was discharged with an indwelling catheter, potassium and phosphorus lowering, furosemide, and erythropoietin. The agreed focus was palliative care for improved life quality and refraining from life-extending interventions.

### 2.1. Outcomes and Follow-Up

Over 6 months (December 2023–May 2024) under Hadassah home hospice service in Jerusalem, the patient received weekly palliative nurse and bimonthly doctor visits. The goal of the patient, family, and staff was to avoid hospital visits. Bimonthly biochemical home blood tests assisted in adjusting medications (potassium and phosphorus binders), and oral furosemide was titrated according to pedal edema and lung auscultation. The indwelling catheter remained, and erythropoietin injections were administered weekly. There was no use of ECG or other monitoring devices other than blood pressure taken by the aide.

This treatment plan proved itself successful. During the reported time, no ER visits or hospitalizations occurred (a 100% reduction from 13 visits over the preceding year). Moreover, the patient's creatinine steadily improved, and a decrease from 7.6 mg/dL in December 2023 to 3.2 mg/dL in May 2024 was observed (58% reduction). GFR improved from 6.7 mL/min/1.73 m^2^ to 18.2 mL/min/1.73 m^2^. Potassium and phosphorus levels stabilized as well. Potassium reduced from 6.1 mEq/L to 3.7–4 mEq/L and phosphorus from 6.2 mg/dL to 3.5–4.3 mg/dL (see Figure 1).

Clinically, the patient exhibited improvement in cognitive function, memory, and communication. Peripheral edema was managed with gradually reduced furosemide, and no pulmonary edema or pericardial effusion due to kidney failure was observed. Overall, quality of life improved significantly as reported by himself and family and observed by the staff.

## 3. Discussion

In the reported case, our elderly patient opted against dialysis despite ESKD and focused on conservative therapy, including the intention to avoid ER visits and hospitalizations given their nonpalliative nature and his desire to be at home. These informed decisions were supported by a skilled palliative staff (doctor, nurse, and social worker) and the patient's family and in-house aide.

The decision appeared to benefit this patient. Initiating hemodialysis may have temporarily improved kidney function, however, associated complications and the burden of frequent dialysis sessions posed significant risks. In contrast, palliative treatment in a home environment allowed for focused care and active patient involvement.

Our case highlights that palliative treatment does not necessarily lead to worsening kidney function or imminent death. On the contrary, significant improvement in kidney function and overall quality of life can be achieved. While this is an isolated case, it underscores the benefits of conservative management in selected elderly ESKD patients in Israel.

## 4. Conclusion

This case report demonstrates that conservative palliative management can lead to significant improvement in kidney function and quality of life in elderly ESKD patients in Israel. Although this phenomenon has previously been recognized in a subset of patients in other places such as the United States [1, 7], Canada [3], or England [9], such treatment requires both patient and family agreement and cooperation in managing treatment at home. A skilled and highly available palliative team is required to enable such results.

Similar results were presented on a larger scale, and not specifically for renal disease, in a recently published JAMA study. The study focused on early (i.e., in the ER) initiation of palliative care patients and showed that such intervention was useful in reducing hospital readmission rates, without having a negative effect on mortality rates, compared to the preintervention period [10].

Despite the noninferior results concerning mortality with early palliative care in selected cases, and the benefit in preventing additional hospital visits, implementing this approach appears challenging for clinicians. A recent 2024 study examining the practice of shared decision-making between nephrologists and ERSD patients in Israel, found only partial implementation of such an approach among physicians and concluded that developing and implementing frameworks for joint and informed decision-making in ESKD patients is necessary and may raise the numbers of patients opting for conservative management [11].

It is important, in conclusion, to report additional Israeli-based cases and nephrologists' approach toward such patients. Such reports support our clinicians and patients who opt for a palliative approach but may fear potential negative results. Furthermore, they will hopefully assist future development of appropriate locally based guidelines for conservative treatment in similar cases. We are aware that a single case report is insufficient for creating guidelines for the palliative management of ESKD in elderly patients, but we hope more such reports will enable this in the future.

## Figures and Tables

**Table 1:** Emergency room and hospital visits.

Dates	ER/hospitalization	Duration in hospital (days)	Where in hospital	Cause of hospital visit	Max creatinine (mg\dL)	Min creatinine (mg/dL)	Max potassium (mEq/L)	Min potassium (mEq/L)	Max phosphorus (mEq/L)	Min phosphorus (mEq/L)
Jan-23	ER	1	ER	Urinary retention	1.32	Null	3.8	Null	Null	Null
Mar-23	ER	1	ER	UTI, urinary catheter obstruction	1.14	Null	5.1	Null	Null	Null
Mar-23	Hospitalization	8	Internal medicine	UTI, urinary catheter obstruction	2.12	1.12	5.4	3.9	2.9	2.7
May-23	Hospitalization	6	Internal medicine (3 days), urology (3 days)	UTI, urosepsis	2.1	1.58	5	3.7	2.7	2.7
May-23	ER	1	ER	Accidental extraction of urinary catheter	Null	Null	Null	Null	Null	Null
Aug-23	ER	1	ER	Acute or chronic kidney injury	3.15	Null	4.3	Null	Null	Null
Sep-23	Hospitalization	3	Urology	UTI, urinary catheter obstruction	5.7	4.4	5.1	4.9	Null	Null
Sep-23	Hospitalization	3	Urology	UTI, macrohematuria	5.06	4.6	5.5	4.6	Null	Null
Oct-23	ER	1	ER	Anemia (HB 6.8), UTI	4.9	Null	5.2	Null	Null	Null
Oct-23	ER	1	ER	Macrohematuria	4.13	Null	4.9	Null	Null	Null
Nov-23	ER	1	ER	UTI, urinary catheter obstruction	6.17	5.6	6.1	4.9	Null	Null
Dec-23	Hospitalization	14	Internal medicine	UTI, acute or chronic kidney injury, initial decision on palliative hospice care	6.8	5.8	5.5	4.2	5.2	5

## Data Availability

The data that support the findings of this study are available from the corresponding author upon reasonable request.
